# Exploratory Evaluation of the *parkrun Practice* Initiative in Australian General Practice: A Cross‐Sectional Survey of Healthcare Professionals and Event Directors

**DOI:** 10.1002/hpja.70260

**Published:** 2026-10-17

**Authors:** Callum Leese, Ruth Eagles, Anne Grunseit, Benni Whiteley, Lewis Boylen, Robert Mann, Emma Cockcroft, Moira Smith

**Affiliations:** ^1^ School of Medicine University of Dundee Dundee UK; ^2^ College of Medicine and Dentistry James Cook University Townsville Australia; ^3^ Faculty of Health University of Technology Sydney Sydney Australia; ^4^ Department of Public Health and Sport Sciences University of Exeter Exeter UK; ^5^ Department of Health and Community Sciences University of Exeter Exeter UK; ^6^ College of Healthcare Sciences James Cook University Townsville Australia

**Keywords:** community, general practice, physical activity, social prescribing

## Abstract

**Issue Addressed:**

The health benefits of physical activity (PA) are overwhelming, yet a high proportion of the Australian population does not meet recommended guidelines. Community‐based initiatives such as *parkrun* offer a scalable approach to increasing PA. The *parkrun practice* initiative links general practice with local *parkrun* events to support people to become more physically active. In the United Kingdom, 32% of primary care centres are ‘parkrun practices’, however, this initiative has yet to be examined in the Australian context. Therefore, the aim of this study was to evaluate the *parkrun practice* initiative within Australian primary care.

**Methods:**

An exploratory cross‐sectional survey was conducted with Australian *parkrun practices* and *parkrun* event directors. Surveys, adapted from a previous UK evaluation tool, captured motivations for participation, approaches to implementation, perceived benefits and barriers. Descriptive statistics were used for quantitative data, whilst free‐text responses were analysed thematically using deductive and data‐driven coding.

**Results:**

Only 1.4% of Australian GP clinics (*n* = 98) are registered *parkrun practices*. Nineteen *parkrun practices* (26.0%) and 140 parkrun event directors (26.1%) participated in the survey. Motivations for practice participation included improving patient wellbeing, personal advocacy of parkrun and community engagement. Reported benefits included improvements in patient wellbeing and increased staff awareness of PA. Barriers to implementation for both practices and event directors included limited awareness of the initiative, lack of time, competing priorities and unclear responsibility for delivery.

**Conclusion:**

The *parkrun practice* initiative in Australia remains in its early stages, with uptake constrained by limited awareness and reliance on voluntary capacity. A collaborative approach between key stakeholders is required to ensure the initiative's potential is achieved.

**So What?:**

Whilst collaborative approaches with third parties to promote PA in primary care have potential, collaboration and co‐design with all stakeholders is required to ensure effective implementation.

## 1 Introduction

There is compelling evidence of the benefits of physical activity (PA) on both physical and mental health [1, 2]. Despite this, 31% of the global population fail to meet the PA guidelines set by the World Health Organisation [3]. The problem is even more pronounced in Australia, with 46% failing to meet the recommended guidelines [4]. Importantly, PA rates are not evenly distributed across populations, with lower levels of activity consistently observed among people living in socioeconomically disadvantaged communities. As a result, physical inactivity both reflects and contributes to widening health inequities.

Given these inequities, healthcare settings are an important context for promoting PA at a population level. The introduction of Chronic Disease Management Plans in 2006 by the Australian Government allows for the provision of PA support for patients with long‐term conditions (referred by GPs) delivered by Exercise Physiologists [5]. Despite this, PA promotion remains limited, with barriers including insufficient health professional engagement and financial constraints [6, 7, 8]. A potential solution to addressing some of these barriers is to better connect healthcare‐based PA promotion with accessible, community initiatives. One such initiative is *parkrun*, a free, weekly timed 5‐km run or walk which began in the United Kingdom (UK) and has since grown into a global phenomenon [9]. *parkrun* is now ubiquitous in many countries, including Australia, where more than 540 events take place across all states and territories, spanning urban and rural settings [10]. The widespread availability suggests that parkrun may offer a scalable community‐based platform for supporting PA participation [11]. In addition, an Australian study reported that *parkrun* participants reported a higher satisfaction with physical health than the general population [12]. Women reported well‐being benefits related to improved mental health, whereas men benefited predominantly through community engagement. These findings suggest that *parkrun* may support not only PA participation but also broader wellbeing and social connection, making it a relevant platform for health promotion and social prescribing initiatives.

Building on the success of *parkrun*, the *parkrun practice* (PP) initiative was launched in the United Kingdom in 2018, followed by The Republic of Ireland (2020) and Australia (2022). This social prescribing initiative aims to connect general practice (GP) clinics with local *parkrun* events (PE) to help people living with long‐term health conditions, as well as practice staff, become more physically active [13, 14]. The initiative seeks to integrate preventative health care within primary care and support in the creation of supportive environments for health promotion, by enabling the individual through advocacy; all key principles of health promotion highlighted in the foundational 1986 Ottawa Charter for Health Promotion [15]. The broader relevance of this approach is reflected in a 2019 RACGP report, which acknowledged the potential value of social prescribing to improve health and well‐being in Australia and made recommendations to integrate it within Australian healthcare systems.

Implementation of the PP initiative in the United Kingdom has shown benefits, including improved patient and staff well‐being, and improved staff morale [16]. Barriers to implementation also exist, notably limited time, variable engagement from practice staff, and limited access to a local *parkrun*. These findings provide a useful foundation, but there is currently no evaluation of the PP initiative in Australia, where different contextual and healthcare system factors may influence implementation and outcomes. Given that the initiative only commenced in Australia in 2022, it remains in its infancy. Therefore, this exploratory study aimed to evaluate the PP initiative from the perspective of both general practices and PE directors, including exploring the motivations for joining, means of delivery and both the perceived benefits and barriers.

## 2 Methods

The PP initiative is voluntary and healthcare led, requiring primary care ‘practices’ to partner with their local *parkrun*. Once this partnership is registered, the practice receives an implementation toolkit and promotional materials from *parkrun* to support various engagement activities, such as patient signposting, staff participation and liaison with local PEs [13]. Whilst the initiative emphasises flexibility and seeks to keep the burden low, long‐term integration relies on localised decision‐making regarding delivery and promotion. An outline of the PP initiative is available via the Royal Australian College of General Practices (RACGP) website [13], and the component activities in Files S1 and S2.

### 2.1 Survey Design and Distribution

We used a survey previously developed by an advisory panel of experts and implemented for evaluation of the UK PP initiative [16]. Minor adaptations (e.g., the UK survey asked about practice ‘list size’ referring to the total number of patients registered at a practice whereas the Australian survey asked instead about Full‐Time Equivalent General Practitioners as a proxy of list size) were made to make the original survey relevant for an Australian audience (see File S3).

PPs in Australia are advertised on the RACGP website. As of October 2025, 98 PPs were listed. Of these, 73 had publicly available email addresses and were in Australia (3 not in Australia, 2 were duplicates, 15 email addresses were not available and 5 available addresses came back ‘undeliverable’). The PP survey was distributed via email by the lead researcher on 21st October 2025, with follow‐up reminders on 5th and 13th November 2025. The survey closed on 18th November 2025, after 4 weeks.

The PE survey was sent to all *parkrun* Australia events via email by the communications team at *parkrun* Australia (*n* = 537). The survey was also shared via *parkrun* social media channels and newsletters.

One representative from each practice or PE was asked to complete the survey which was administered via an online platform (JISC Online Surveys). Informed consent was obtained prior to participation in the survey. Participation was voluntary and no remuneration was provided. Ethics approval for non‐clinical research was gained from respective universities (UOD‐SMED‐SLS‐Staff‐2024‐24‐90 and 25H‐0327).

### 2.2 Participants

Only *parkrun* event directors (EDs) or employed staff (both clinical and non‐clinical) of accredited PPs in Australia were eligible to participate.

### 2.3 Data Analysis

Data were downloaded and cleaned in Microsoft Excel (Version 2402). Descriptive statistics were used to present demographic data, closed question responses and Likert scale question responses. Survey responses for the free‐text questions were analysed as outlined in previously published work [16, 17]. Coding was both deductive (informed by a framework from prior work) and data‐driven, allowing for new insights in this Australian context. Generated codes were categorised into themes, with a frequency analysis of generated themes then conducted.

## 3 Results

### 3.1 Sample Characteristics

At the time of survey distribution, there were 98 registered PPs and 532 PEs in Australia. The 98 PPs represent approximately 1.4% of all primary care centres (*n* ~ 7000) [13, 18]. In total, 159 respondents (19 PP, 140 PE) consented and completed the survey.

### 3.2 Parkrun Practices

Responses from 19 healthcare workers (HCW), each from a different site, were included in the final analysis. This represented 26.0% of all PPs at the time the survey was disseminated. Practice and HCW demographics are shown in Table 1.

**TABLE 1:** Practice and respondent characteristics (*n* = 19).

Practice characteristics	*n* (%)
Practice population	
< 4000	7 (36.8%)
4000–7999	6 (31.6%)
8000–11 999	2 (10.5%)
12 000–15 999	2 (10.5%)
16 000–20 000	0 (0%)
> 20 000	2 (10.5%)
Number of parkrun events linked with each responding practice	
1	17 (89.5%)
2	1 (5.3%)
3 or more	1 (5.3%)
Length of affiliation	
< 1 year	2 (10.5%)
1–2 years	4 (21.1%)
2–3 years	7 (36.8%)
4 or more years	6 (31.6%)
Establishing links with parkrun	
parkrun event team made first contact	4 (21.1%)
Practice made first contact	13 (68.4%)
Other	2 (10.5%)

Healthcare respondent characteristics	*n* (%)
Responder role	
General practitioner	11 (57.9%)
Nurse	3 (15.8%)
Practice manager	3 (15.8%)
Administration team	0 (0%)
Doctor in training	0 (0%)
Physiotherapist	1 (5.3%)
Other	1 (5.3%)
Responding staff member a parkrun participant prior to establishing the initiative	
Yes	16 (84.2%)
No	3 (15.8%)

### 3.3 Event Directors

One‐hundred and forty EDs were included in the analysis, representing 26.1% of all PEs in Australia. Of responding EDs, 135 reported that their event was not linked with a local GP practice. Eight (5.9%) reported having tried unsuccessfully to link with a GP practice. PEs can be linked with one or more GP practices, and practices can be linked with one or more events. Five events reported that their event was linked to at least one GP practice; four were affiliated with a single local practice (*n* = 4, 80%), whilst one event was linked with two practices (*n* = 1, 20%).

### 3.4 Motivation for HCW's Signing‐Up

Alongside improving patient wellbeing (58.0%), personal advocacy of *parkrun* participation (47.4%) and staff wellbeing (10.5%), respondents highlighted community engagement as a motivation for engaging in the initiative (31.6%). Of respondents, 84.2% (*n* = 16) reported prior participation in parkrun, with this appearing to be a large influence on practices obtaining PP status:

As an avid parkrunner myself I jumped into the prospect of parkrun practice and contacted my local parkrun organiser and went with staff for coffee or breakfast afterwards. (HCW004)

### 3.5 Implementation

Table 2 shows HCWs reported delivering the initiative to patients and/or carers most commonly through regular discussions (52.6%) and sharing flyers (31.6%). Delivery to staff was primarily by regularly attending parkrun (89.5%) and accompanying colleagues (52.6%) (Table 2).

**TABLE 2:** Activities adopted by parkrun practices (*n* = 19) to promote the initiative.

Theme	% (*n*)
Activities adopted by parkrun practices to ‘promote the initiative to patients and carers’	
Regularly speaking to patient ± carers about parkrun and the benefits of participating	52.6% (10)
Regularly sharing parkrun flyers (hard copy or digital)	31.6% (6)
Using text‐based messaging software to suggest parkrun to patients	0
Presence of a parkrun noticeboard in the practice	21.1% (4)
Delivering a parkrun practice slideshow on practice TV screens	15.8% (3)
Delivering a presentation to patients/carers about parkrun and its benefits	0
Hosting information about parkrun on the practice website	15.8% (3)
Highlighting parkrun in practice newsletters	10.5% (2)
Sharing parkrun and stories on social media platforms	21.1% (4)
Organising a day for staff and patients to attend a local parkrun together	21.1% (4)
Encouraging other initiatives that link with parkrun, like 5 k Your Way	26.3% (5)
Other	47.4% (9)
Not known	0
Activities adopted by parkrun practices to ‘promote the initiative to *staff*’	
Staff regularly attending parkrun	89.5% (17)
Practice‐wide presentation on parkrun	31.6% (6)
Sharing of case studies or practices that have linked with parkrun	10.5% (2)
Staff accompanying colleagues to parkrun events	52.6% (10)
Presence of a parkrun staff noticeboard	5.3% (1)
Staff wellbeing challenges that reward parkrun participation	10.5% (2)
Staff hosting a volunteer take‐over of the parkrun event	42.1% (8)
Sharing inspiration stories on their networks and wider	10.5% (2)
Other	15.8% (3)
Not known	10.5% (2)

EDs were asked what activities they witnessed being undertaken by the GP practice. When comparing the responses from the five *parkrun* EDs that reported being paired with a primary care centre with the responses from 19 different primary care centres, HCWs reported a greater range of promotion activities compared with EDs. All 5 EDs reported just 5 activities being undertaken, compared to 10 different activities reported by HCWs. Given the lack of response from EDs that were paired with a practice the result must be interpreted with caution, but they appear to highlight a discrepancy between what activities are reportedly being undertaken by practices to deliver the initiative, and those that EDs perceive are being delivered.

Almost 90% of HCW reported that staff in their practice regularly take part in their local PE, with 52.6% reporting that HCW regularly discuss *parkrun* with patients and 31.6% reporting regularly sharing written information with patients and/or carers (as shown in Figure 1).

**FIGURE 1:**
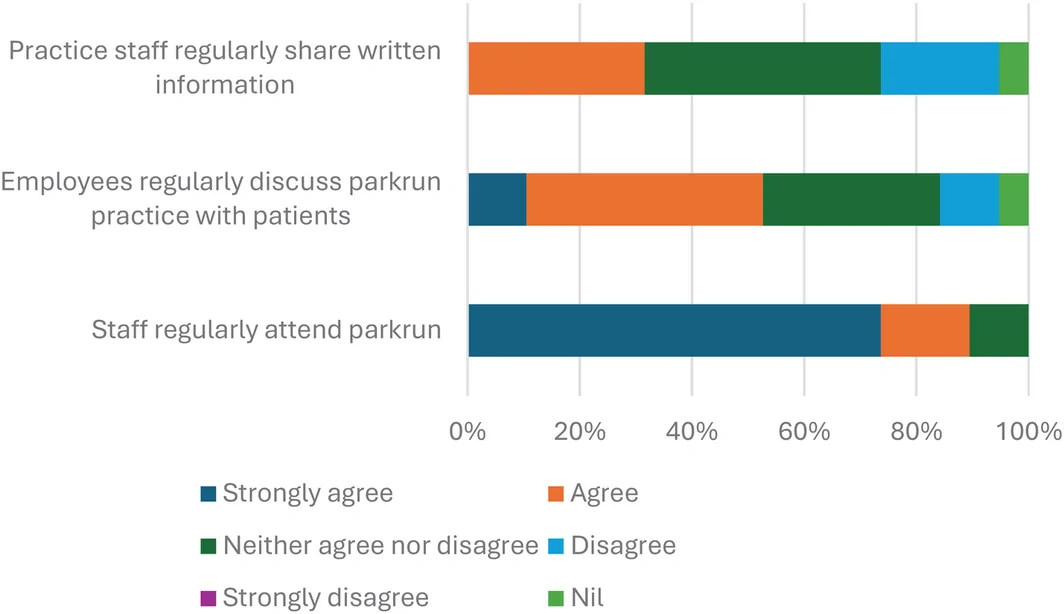
Agreement with implementation of key elements of the parkrun practice initiative, as perceived by HCW (*n* = 19) in the parkrun survey (5‐point Likert scale).

### 3.6 Impact

HCW highlighted two main benefits of the PP initiative. These were: (a) improvements in patient health and wellbeing (36.8%); and (b) improved staff awareness of the benefits of PA (15.8%).

I have seen patients join and how their lives are changed for the better: weight loss, increased fitness, community engagement and improved sense of well‐being are some of the reported positive impacts. (HCW008)

Increases awareness of social prescribing amongst GPs and other health workers. (HCW001)

### 3.7 Challenges

The content analysis showed EDs experienced several challenges to the delivery of the PP initiative (Table 3).

**TABLE 3:** A summary of the qualitative content analysis for the survey assessing the ‘challenges experienced by event organisers’ in trying unsuccessfully to implement the parkrun practice initiative (*n* = 135).

Theme	Codes included	Percentage of responses (*n* = 135)	Illustrative quotes
ED limitations	Lack of awareness/understanding regarding parkrun practice initiative	62.2% (*n* = 84)	‘Didn't know about it.’ ED140 ‘didn't know this was possible’. ED131
	Time/Workload	9.6% (*n* = 13)	‘ED's are volunteer roles with limited time’. ED128
	Not role of ED	6.7% (*n* = 9)	‘The core focus has been on establishing and sustaining our core volunteer crew and running a sustainable event. Given that the event is run 100% by volunteers that have day jobs and other lives, doing any work to partner with a GP practice has not been at the forefront of our planning’. ED111
Communication:	Difficulty establishing ongoing communication	3.7% (*n* = 5)	‘We did a formal announcement during the welcome brief and they delivered a speech and introduction of our local GPs including several who are active members of our parkrun community, however nothing further has happened since’. ED139
	Knowing who to contact	4.4% (*n* = 6)	‘There are so many in our area which one do you choose!’ ED135
Structure of the initiative	Lack of support/guidance	8.1% (*n* = 11)	‘No specific guidance on how best to do this’. ED42
	Organisational constraints	2.2% (*n* = 3)	‘I feel that is more the role of Parkrun HQ to drive nationally, rather than on an event basis’. ED40

Abbreviation: ED = event director.

The three most frequently cited challenges were: (a) a lack of awareness of the initiative (62.2%); (b) a lack of time of already stretched volunteers to deliver the initiative (9.6%); and (c) a lack of awareness of support/guidance (8.1%).

Not considered it yet. I'm not sure if health prescribing is much of a thing in Australia. EO138

Our parkrun has been running for just nearing 2 years. The core focus has been on establishing and sustaining our core volunteer crew and running a sustainable event. Given that the event is run 100% by volunteers that have day jobs and other lives, doing any work to partner with a GP practice has not been at the forefront of our planning. And to be honest, it should work the other way and that GP practices should go out of their way to partner with their local parkrun. EO111

We have not been given any direction from parkrun Australia on how best to go about this. EO011

HCW cited similar challenges (Table 4), including: (a) a lack of engagement from colleagues and patients (42.1%); and (b) a lack of time to deliver the initiative (21.1%). A lack of engagement cited by practice staff was related to the challenges of: (a) convincing staff of the benefits both for themselves and those they care for; and (b) the ongoing ‘buy‐in’/enthusiasm to ensure longer term successful delivery.

Resistance from one of the GPs who is quite anti exercise for herself! HCW05

Some practitioners not offering parkrun as they forget it is an option. HCW07

Just finding time to promote and remembering it as an option. HCW13

**TABLE 4:** A summary of the qualitative content analysis for the survey assessing the challenges experienced by healthcare workers in the implementation of parkrun practice initiative (*n* = 19).

Theme	Codes included	Percentage of responses (*n*)	Illustrative quotes
Constraints	Engagement	42.1% (*n* = 8)	‘Some practitioners not offering parkrun as they forget it is an option’. (HCW06)
	Time/workload	21.1% (*n* = 4)	‘Just finding time to promote’. (HCW13)
Preconceptions and understanding:	Challenging preconceptions	5.2% (*n* = 1)	‘At times patients are still contemplating joining or has misgivings about joining an event with “run” in the title despite clear explanation’. (HCW08)
Structure of the initiative	Organisational constraints	10.5% (*n* = 2)	‘There is a lack of support from RACGP.’ (HCW01) ‘we wanted to sponsor some local events but were prevented due to national marketing arrangements.’ (HCW09)
External factors	Geography	10.5% (*n* = 2)	‘disabled clients and hilly primary parkrun’. (HCW12)

Abbreviations: HCW = healthcare worker; RAGCP = Royal Australian College of General Practitioners.

## 4 Discussion

Our survey of 19 practices and 140 parkrun EDs showed that despite enthusiasm for the initiative, EDs and practices experience challenges in establishing and promoting the PP initiative in Australia. Compared to the United Kingdom with more than 2000 PPs representing more than 32% of all primary care centres [19, 20], the uptake is comparatively low in Australia (*n* = 98), approximately 1.4% of all primary care centres [13, 18]. Motivations for adoption and subsequent implementation activities were similar in the current study compared with the recent UK evaluation [16]. However, a much wider range of perceived benefits was reported in the United Kingdom, likely reflecting the greater numbers of practices involved and the flexibility to tailor implementation to specific locations.

We hypothesise that the comparatively limited uptake of the initiative in Australia reflects contextual and structural factors rather than low commitment from clinicians or stakeholders. Specifically, the initiative was launched in 2022 at a time of unprecedented pressure on general practice due to the COVID19 pandemic, and early dissemination was intentionally conservative to avoid placing additional burden on GPs. In addition, the Australian primary care landscape differs from that of the United Kingdom, with multiple professional colleges and less routine exposure to concepts such as social prescribing. Funding models may influence the services provided by GPs. In Australia, general practice is funded in a fee‐for‐service model, whereby each patient attendance attracts a government payment via the Medicare Benefits Schedule (MBS) [21]. This payment may form part or all of the service payment. Different payment item numbers are available for different services. This model has the potential to incentivise a high volume of appointments and may discourage preventive health care [21]. There is currently no dedicated item number for providing social prescribing, meaning that social prescriptions, such as a discussion of parkrun, must be integrated into another type of service (such as a routine consultation as a component of the management plan, or as part of a chronic disease care plan) to attract funding. With multiple competing priorities and no dedicated Medicare item number, social prescriptions such as parkrun may be less likely to be initiated. Together, these factors may have constrained early awareness and engagement, but signal opportunities for future collaboration, education and more coordinated implementation. In the United Kingdom, although not direct to GP practices, funding is available to support physical activity promotion. Social prescribing is well established nationally, with link workers or referral pathways often established in primary care settings [22].

Interestingly, the greatest barrier to implementation in the United Kingdom was a lack of time, whereas in Australia lack of time was second to lack of engagement/awareness. Disengagement in voluntary implemented initiatives in healthcare is complex and often multi‐factorial. Competing clinical and organisational priorities may result in capacity pressures, with additional voluntary working being perceived as non‐core [23]. Furthermore, research into social prescribing highlighted that engagement often relied on belief in the benefit, and that historically heterogenous and methodologically weak research resulted in limited clinician ‘buy‐in’ [24]. It is plausible that similar sentiments exist around the promotion of physical activity in primary care. The research by Pescheny and colleagues [24] also highlighted that social prescribing models suffer from unclear responsibility, with ambiguity between clinicians, link workers and voluntary sector partners reducing engagement from all groups.

Despite this, the PP initiative remains relevant to Australian health policy. The National Preventative Health Strategy 2021–2030 [25] identified a need to support health professionals to promote PA and social prescribing whilst also calling for improvements in connection between communities and health services. Our research highlights that practice staff identified community engagement as one of the motivators for joining the initiative, with the previous research in the United Kingdom [16] highlighting community engagement as one of the key benefits. Alongside national policy, the PP initiative also aligns with the RACGP priorities, namely engagement with social prescribing [26]. Collaborative support and engagement from stakeholders such as GP colleges and all levels of government could raise the profile of the PP initiative whilst supporting policy agendas and addressing lack of awareness. Enhanced delivery could be supported by engaging the practices' wider multidisciplinary team, with nurses, physiotherapists, or practice managers potentially better placed to lead implementation. Financial remuneration systems for time spent on social prescribing initiatives, such as PP, may also promote uptake [27].

Currently, the initiative is implemented on a voluntary basis. Practices in lower socio‐economic settings face resourcing deficits and higher demand, rendering the equitable implementation of voluntary programmes difficult [28]. The reverse of this is also true: unfunded, volunteer‐reliant models inherently favour affluent clinics with spare capacity. Individuals who live in lower socio‐economic communities are less likely to be active [29]; any initiatives' failure to reach these groups risks further widening health inequities. Despite parkrun being more accessible in areas of higher socio‐economic status (SES) [30] growth in parkrun participation is steeper in lower socio‐economic areas [31]. A recent publication has highlighted that PP*s* in England are more likely to be located in affluent areas and serve populations with a higher proportion of older, white patients [32]. Therefore, there is scope for a strengthening of the PP initiative to take a health equity lens, including additional support for initiatives in areas that may benefit most.

## 5 Limitations

This survey provides insight into the implementation of the initiative across Australia; however, several limitations should be acknowledged. Response rates were low, which may have led to responder bias, limited adjusted/multiple variable analyses and the generalisability of the findings. In particular, the number of responses from *parkruns* which reported being linked to a practice was very low (*n* = 5), making comparisons with practice responses unreliable.

The survey included free‐text questions, which generating additional insights, particularly regarding perceived barriers and benefits of the initiative. Nonetheless, responses were often brief and lacked depth. A more comprehensive understanding of these experiences could be achieved via more in‐depth qualitative research (e.g., focus groups).

Demographic information was limited, restricting examination of differences across geographic contexts, such as between states of Australia or across urban, rural and remote areas. Despite these limitations, the survey provides an important preliminary overview of engagement with the initiative across a range of Australian settings and identifies key areas for further in‐depth exploration.

## 6 Conclusion

The PP initiative has not yet been promoted or implemented in a systematic or coordinated manner in Australia, with uptake to date occurring primarily through organic engagement, in part reflecting its voluntary nature. This has constrained its reach and effectiveness despite its demonstrated potential to improve health and wellbeing of staff and patients. A collaborative and actively resourced dissemination strategy involving GP colleges, government and parkrun Australia is required to enable the initiative to achieve its full potential.

## Funding

The authors have nothing to report.

## Ethics Statement

Ethics for research was sought and approved initially by the University of Dundee's Research Ethics Committee (UOD‐SMED‐SLS‐Staff‐2024‐24‐90) and then by James Cook University ethics (25H‐0327).

## Conflicts of Interest

The authors declare no conflicts of interest.

## Supporting information


**File S1:** Recommended activities for *parkrun practice*: Patient and carer engagement.
**File S2:** Recommended activities to promote parkrun within the practice team.
**File S3:** parkrun practice survey.

## Data Availability

The data that support the findings of this study are available from the corresponding author upon reasonable request.
