# Supplementary material for: Exploratory Evaluation of the parkrun Practice Initiative in Australian General Practice: A Cross‐Sectional Survey of Healthcare Professionals and Event Directors
Source: Health Promot J Austr. 2026 Oct 17;38(1):e70260. doi: 10.1002/hpja.70260 (PMC13675718; doi:10.1002/hpja.70260)
Supplement: Supplementary file 1 [file HPJA-38-0-s001.docx]

**Supplementary File 1: Recommended Activities for parkrun Practice: Patient and Carer Engagement**

While participation in parkrun practice activities is optional, parkrun recommends the following approaches to help promote the initiative to patients and carers:

1. Discussing parkrun regularly with patients and/or carers, including the potential benefits of taking part.
2. Sharing parkrun promotional materials, either as printed leaflets or digital resources.
3. Using text‑based messaging systems (e.g., practice SMS services) to suggest parkrun to suitable patients.
4. Displaying a dedicated parkrun noticeboard within the practice.
5. Showing a parkrun practice slideshow on waiting-room or practice TV screens.
6. Delivering educational presentations to patients and/or carers about parkrun and its health benefits.
7. Including information about parkrun on the practice website.
8. Highlighting parkrun in practice newsletters or similar communications.
9. Sharing parkrun updates and stories through the practice’s social media channels.
10. Organising a practice-supported attendance day, where staff and patients join a local parkrun together.
11. Promoting related initiatives, such as *5k Your Way*, that complement parkrun participation.

**Supplementary File 2: Recommended Activities to Promote parkrun Within the Practice Team**

To encourage engagement among practice staff, the following activities are recommended:

1. Staff regularly taking part in parkrun events (running, walking, or volunteering).
2. Delivering a practice-wide presentation to inform staff about parkrun and its benefits.
3. Sharing case studies or examples from other practices successfully linked with parkrun.
4. Staff attending parkrun events together, supporting colleagues who are new to it.
5. Maintaining a staff-focused parkrun noticeboard within the practice.
6. Running staff wellbeing challenges that acknowledge or reward parkrun participation.
7. Organising a practice-led volunteer takeover at a local parkrun event.
8. Sharing inspirational parkrun stories through internal networks or more widely.

**Supplementary File 3: parkrun Practice Survey**

Project Title: Australia parkrun Practice Evaluation

**Parkrun Practice Evaluation Consent Form**

You are being invited to take part in a research study. Before you decide to take part, it is important for you to understand why the research is being done and what it will involve. Please take the time to read the following information carefully and discuss it with others if you wish. If there is anything that is not clear or if you would like more information, please ask us (contact details below). Take time to decide whether you wish to take part, it is entirely up to you. Thank you for reading.

1. Who is conducting the research?

The principal investigator I (*removed for anonymity*) of the (*removed for anonymity*), email: (*removed for anonymity*). He is working alongside researchers from the (*removed for anonymity*).

2. Who is funding the study?

There is no funding involved in this study.

3. What is the purpose of the study?

The purpose of the study is to understand what health care professionals think about the *parkrun practice* initiative. Specifically, this study will explore how the *parkrun practice* initiative is being implemented in primary care, the motivation behind its adoption, challenges to its implementation, and how it could be improved.

4. Do I have to take part?

No. Taking part is entirely voluntary; choosing not to take part will not disadvantage you in any way and you are free to withdraw from the study at any time without explanation and without penalty. You can exit the survey at any time and your responses will not be submitted. Once completed, if you wish your response removed, please email (*removed for anonymity*). Due to the anonymisation, please provide date and time of completion, with further information possibly required at the request of the principal investigator to ensure deletion of correct data.

5. What will happen to me if I take part?

If you volunteer to take part in this study, you will be asked to complete a survey that will take approximately 5 minutes to complete. The survey will be done online and will ask about your experiences of the parkrun practice initiative. The survey will be recorded on JISC online survey tool. There is no reward or payment for participation.

6. How do I consent to taking part?

Please check (tick) the box and continue to completion of the survey.

7. What will happen to me if I take part?

If you volunteer to take part in this study, you will be asked to complete a survey that will take approximately 5 minutes to complete. The survey will be done online, and you can exit at any time.

8. What are the possible disadvantages of taking part?

There are no envisaged disadvantages to taking part.

9. What are the possible benefits of taking part?

You can benefit from taking part in the study by influencing the intended development of the *parkrun practice* initiative; to aid you and other healthcare professionals implement it more effectively in the future.

10. Will my taking part in the study be kept confidential?

Yes, confidentiality will be maintained, with no personal data collected within the survey. Data regarding clinical specialties and location of work will not be presented or linked to individual level data and will only be presented after collation and analysis. Each data set will be allocated a non-identifiable participant number that will be used when labelling quotations.

11. What will happen to the information I provide?

All data are kept in confidence and will be stored on a password protected system (*removed for anonymity*). Data will be held for a minimum period of 5 years. Upon completion of this period or publication of the data, identifying information will be deleted and destroyed by (*removed for anonymity*).

Results will be shared with parkrun in the form of a short report and made publicly available. Research will also be published in a peer-reviewed journal. Participants will be able to access future published work through Discovery, (*removed for anonymity*) repository, or by request to the researcher, subject to any funder or publisher requirements.

**Data Protection**

The personal data that will be collected and processed in this study are job role and region of current employment.

The University asserts that it is lawful for it to process your personal data in this project as the processing is necessary for the performance of a task carried out in the public interest or in the exercise of official authority vested in the controller.

The (*removed for anonymity*) is the data controller for the personal and/or special categories of personal data processed in this project .

The University respects your rights and preferences in relation to your data and if you wish to update, access, erase, or limit the use of your information, please let us know by emailing (*removed for anonymity*)*.* Please note that some of your rights may be limited where personal data is processed for research, but we are happy to discuss that with you. If you wish to complain about the use of your information please contact the University’s Data Protection Officer in the first instance (*removed for anonymity*)). You may also wish to contact the Information Commissioner’s Office (<https://ico.org.uk/>).

You can find more information about the ways that personal data is used at the University at: (*removed for anonymity*)

**Is there someone else I can complain to?**

If you wish to complain about the way the research has been conducted please contact the Convener of the University Research Ethics Committee (*removed for anonymity*). You have the option to email the Convener or leave a voicemail.

**Alternative formats**

*Should you require a copy of the Participant Information Sheet and Consent Form in alternative formats (e.g. large print, Braille) please contact the principal investigator.*

**Please tick the box to confirm you have read the participation information sheet and give consent to participate in the research ☐**

Are you an event organiser or do you work in healthcare? If you are both, please complete the healthcare worker survey and contact us separately via email.

Healthcare Worker (Stem 1)

Event organiser (Stem 2)

**Stem One**

1. What is your current role?

GP

Nurse

Practice Manager

Administration team

GP registrar

Physiotherapist

Other [FREE TEXT]

2.How long have you been a *parkrun practice*?

<1 year

1-2 years

2-3 years

2.5 Is the individual who established your *parkrun practice* status still working within your medical centre?

Yes

No

3. Did you participate in parkrun personally, before your practice joined the initiative?

Yes

No

Can’t remember

4.How many FTE GPs does the practice have (approximately)?

1

2

3

4

5 or more

5.How many parkruns are linked to the practice?

1

2

3

4 or more

7.What motivated the practice to register as a *parkrun* *practice*? *[FREE TEXT]*

8.Regarding making the first links with the local parkrun, how did this happen?

Parkrun event made first contact

Practice made first contact

Other [FREE TEXT]

8.5 Were links made in the same way when pairing with subsequent parkruns?

Yes

No

9.For the following statements please indicate your level of agreement: .

1. The *parkrun practice* initiative has led to improved patient health outcomes.

Strongly agree

Agree

Neither agree nor disagree

Disagree

Strongly disagree

2. The *parkrun practice* initiative has improved staff health and wellbeing.

Strongly agree

Agree

Neither agree nor disagree

Disagree

Strongly disagree

10.What activities have you done to enthuse and engage practice staff about parkrun? (select one or more)

Practice-wide presentation on parkrun

Share case studies of practices that have linked with parkrun

Accompany colleagues to parkrun events

Have a parkrun staff noticeboard

Organize staff wellbeing challenges that reward parkrun participation

Host a volunteer take over of a parkrun event

Share inspirational stories on their networks and wider

Other [FREE TEXT]

No activities undertaken

11.What activities do you do to engage patients/carers about parkrun? (select one or more)

Use text-based messaging software to suggest parkrun to patients

Have a parkrun noticeboard in the practice

Deliver parkrun practice slideshow on practice TV screens

Deliver a presentation to patients/carers about parkrun and its benefits

Host information about parkrun on your practice website

Highlight parkrun in practice newsletters

Share parkrun and stories on social media platforms

Organise a day for staff and patients to attend a local parkrun together

Encourage other initiatives that link with parkrun, like 5k Your Way

Other [FREE TEXT]

No activities undertaken

12 . To what extent do you agree with the following statements?

1. A member of our practice regularly attends the local parkrun.

Strongly agree

Agree

Neither agree nor disagree

Disagree

Strongly disagree

Don’t Know

2. Practice employees regularly discuss parkrun with patients and carers.

Strongly agree

Agree

Neither agree nor disagree

Disagree

Strongly disagree

Don’t Know

3. We regularly share flyers, newsletters or social media posts promoting parkrun

Strongly agree

Agree

Neither agree nor disagree

Disagree

Strongly disagree

Don’t Know

13. Have you experienced any particular challenges in the delivery of the parkrun practice initiative? [FREE TEXT]

14.If willing, could you expand on any perceived positive impacts of the initiative? [FREE TEXT]

**Stem Two**

1 .Is the parkrun event you organise paired with a GP practice?

Yes Question 2

No Question 8

**Stem 2.1**

**2.** Is the individual who established the link with the *parkrun* still an event organiser?

Yes

No

2.If so, how many GP practices are linked to your parkrun?

1

2

3

4 or more

3.Regarding making links with the first local GP practice, how did this happen?

Parkrun event made first contact

Practice made first contact

Other

3.5 Were links made in the same way when linking with subsequent GP practices?

Yes

No

4.For the following statements please indicate your level of agreement: .

1. The *parkrun practice* initiative has had a positive impact on the parkrun event.

Strongly agree

Agree

Neither agree nor disagree

Disagree

Strongly disagree

2. The *parkrun practice* initiative has improved attendance at the parkrun event.

Strongly agree

Agree

Neither agree nor disagree

Disagree

Strongly disagree

5.Are you aware of your linked GP practice(s) engaging staff about parkrun in any of the following (select on or more)

Staff attending parkrun

Practice-wide presentation on parkrun

Sharing of case studies of practices that have linked with parkrun

Staff accompanying colleagues to parkrun events

Presence of a parkrun staff noticeboard in the GP practice

Staff wellbeing challenges that reward parkrun participation

Staff hosting a volunteer take over of the parkrun event

Sharing of inspirational stories on their networks and wider

Other [FREE TEXT]

Not aware of anything

6.Are you aware of the linked GP practice engaging patients/carers about parkrun in any of the following (select one or more)

Speaking to patients/carers about parkrun and the benefits of participating

Sharing parkrun flyers (hard copy or digital)

Using text-based messaging software to suggest parkrun to patients

Presence of a parkrun noticeboard in the practice

Delivering a parkrun practice slideshow on practice TV screens

Delivering a presentation to patients/carers about parkrun and its benefits

Hosting information about parkrun on the practice website

Highlighting parkrun in practice newsletters

Sharing parkrun and stories on social media platforms

Organising a day for staff and patients to attend a local parkrun together

Encouraging other initiatives that link with parkrun, like 5k Your Way

Other [FREE TEXT]

Not aware of anything

7.Are there any particular challenges to engaging and partnering with GP practices? [FREE TEXT]

**Stem 2.2**

8.Have you tried to partner with a GP practice?

Yes

No (go to Q10)

9.If yes, what barriers or challenges have there been to partnering?

10. If no, is there a reason why not?

**Final Page**

Thank you for your participation in this survey assessing the parkrun practice initiative. Your comments will help us as we look to improve the initiative. Please remember to click submit to complete the survey.

The following edits were made from the original survey published in 2025(1):

- The UK survey asked about list size but following discussion with authors MS and RE it was felt this didn’t translate well to the Australian health service, therefore FTE GPs was asked instead as a proxy of practice size (question 4, stem 1)

**REFERENCES**

1.	Leese CJ, Clarke R, Mann RH, Cross R, Al-Zubaidi H, Smith BH, et al. Motivations, delivery, perceived benefits, and barriers to delivery of the parkrun practice initiative in general practices across the UK: a national cross-sectional online survey of healthcare professionals and event organisers. BMC Primary Care. 2025;26(1):137.
